# On the Chemical Stabilities of Ionic Liquids

**DOI:** 10.3390/molecules14093780

**Published:** 2009-09-25

**Authors:** Subbiah Sowmiah, Venkatesan Srinivasadesikan, Ming-Chung Tseng, Yen-Ho Chu

**Affiliations:** Department of Chemistry and Biochemistry, National Chung Cheng University, 168 University Road, Chia-Yi, 621 Taiwan

**Keywords:** ionic liquid, imidazolium, pyridinium, phosphonium, quaternary ammonium, chemical stability

## Abstract

Ionic liquids are novel solvents of interest as greener alternatives to conventional organic solvents aimed at facilitating sustainable chemistry. As a consequence of their unusual physical properties, reusability, and eco-friendly nature, ionic liquids have attracted the attention of organic chemists. Numerous reports have revealed that many catalysts and reagents were supported in the ionic liquid phase, resulting in enhanced reactivity and selectivity in various important reaction transformations. However, synthetic chemists cannot ignore the stability data and intermolecular interactions, or even reactions that are directly applicable to organic reactions in ionic liquids. It is becoming evident from the increasing number of reports on use of ionic liquids as solvents, catalysts, and reagents in organic synthesis that they are not totally inert under many reaction conditions. While in some cases, their unexpected reactivity has proven fortuitous and in others, it is imperative that when selecting an ionic liquid for a particular synthetic application, attention must be paid to its compatibility with the reaction conditions. Even though, more than 200 room temperature ionic liquids are known, only a few reports have commented their effects on reaction mechanisms or rate/stability. Therefore, rather than attempting to give a comprehensive overview of ionic liquid chemistry, this review focuses on the non-innocent nature of ionic liquids, with a decided emphasis to clearly illuminate the ability of ionic liquids to affect the mechanistic aspects of some organic reactions thereby affecting and promoting the yield and selectivity.

## 1. Introduction

Ionic liquids (ILs) are low-melting molten salts composed entirely of ions, and many of them are liquids at room temperature [1,2,3,4,5,6]. These novel solvents are attracting interest as greener alternatives to conventional organic solvents with the aim of facilitating sustainable chemistry. Room temperature ionic liquids (RTILs), often referred to as ‘designer solvents’, have been the great focus of scientists in various fields since they can be tuned for specific applications [7,8,9,10,11]. Promising diverse applications of RTILs continue to expand significantly due to their unusual physical and chemical properties like high thermal stability, lack of inflammability, low volatility, chemical stability and excellent solubility with many organic compounds. Thus, these are considered to be emerging green solvents and potential alternatives to the classical volatile organic solvents. In addition to being “green” solvents [12,13,14], ILs have been used for a myriad of applications in diverse synthetic reactions [15,16,17,18], separations and extractions [19,20,21,22], and electrochemical [23,24], nanotechnological [25,26,27,28], biotechnological [29], and engineering [30,31,32] processes.

The perception that all ionic liquids are “green solvents” may lead to inappropriate experimental design and utilization of these chemicals. As reviewed by Song *et al*., switching from an organic solvent to an ionic liquid often results in a significant improvement in catalytic performance *e.g*. rate acceleration, (enantio)selectivity improvement and an increase in catalyst stability [33,34,35], but synthetic chemists cannot ignore the stability data and intermolecular interactions (or even reactions) that are directly applicable to organic reactions in ionic liquids.

It is usually assumed that these ionic liquids are entirely innocent and non-coordinating solvents. However, such innocuous behavior was not always observed and often led to ‘undesirable’ transformations in reactions [36,37]. The lack of detailed knowledge on their structures and physical-chemical properties requires more attention and certain degree of caution should be exercised when ionic liquids are chosen as solvents.

Even though more than 200 room temperature ionic liquids are known, only a few reports evidence their effects on reaction mechanisms or rate/stability. It is important to note that although many IL-like organic salts have important industrial and commercial applications, the environmental fate and any potential toxicity issues for most ionic liquids are not known, initial data are only now being determined [38]. This review, rather than attempting to give a comprehensive overview of IL chemistry, is focused on the non-innocent nature of ILs, with a decided emphasis on clearly illuminating the ability of ILs to affect the mechanistic aspects of some organic reactions thereby affecting and promoting the yields as well as stereoselectivity.

## 2. Scope of This Review

**Figure 1:**
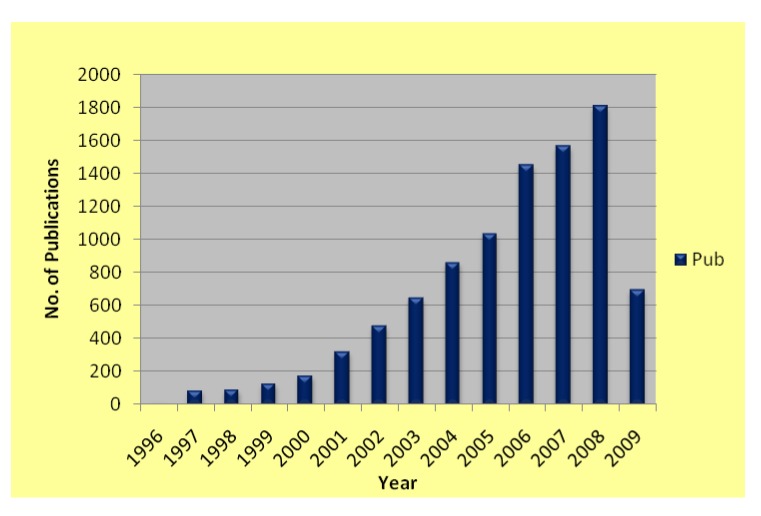
IL publications (on May 27, 2009) determined from the ISI Web of Science in the last fourteen years.

ILs are advanced, technological solvents that can be tailor-synthesized to fit well to a particular application. This emerging field has been extensively reviewed by a number of renowned chemists, including Welton [7], Holbrey [39], and Seddon [40]. Recently, uses of ILs as solvents for chemical synthesis have also been carefully studied and thoroughly reviewed [1,2,3,4,5,6,7,8,9,10,11,41,42,43,44]. There is no doubt that this area of research has been a focal point of green chemistry for the past two decades (Figure 1).

The first room temperature ionic liquid [EtNH_3_][NO_3_] (m.p. 12 °C) was discovered in 1914 [45], but interest did not develop until the discovery of binary ionic liquids made from mixtures of aluminum(III) chloride and *N*-alkylpyridinium [46] or 1,3-dialkylimidazolium chloride [47]. A major drawback of all chloroaluminate(III) ionic liquids was their moisture sensitivity. In 1992, Wilkes and Zawarotko [48] prepared and characterized air and water stable 1-ethyl-3-methylimidazolium based ionic liquids incorporating different anions. Over the years that followed, new classes of cations and anions have been reported. The common cations and anions so far involved in ionic liquids are given below.


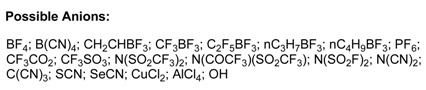



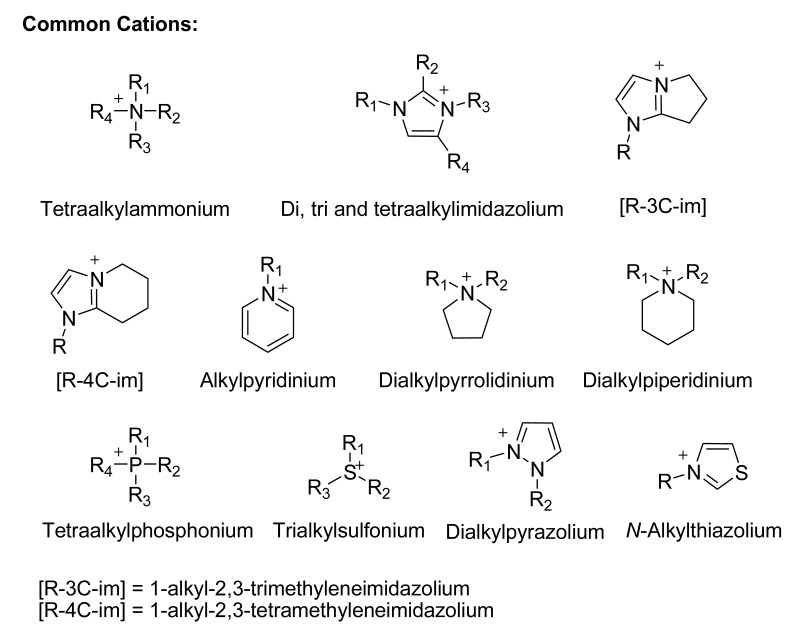


**Table 1:** Comparison of ionic liquids with organic solvents [49].

Property			Organic Solvents		Ionic Liquids
Number of solvents			>1,000		>1,000,000
Applicability			Single function		Multifunction
Catalytic ability			Rare		Common and tunable
Chirality			Rare		Common and tunable
Vapour pressure			Obeys the Clausius-Clapeyron Equation		Negligible under normal conditions
Flammability			Usually flammable		Usually nonflammable
Solvation			Weakly solvating		Strongly solvating
Tunability			Limited range of solvents available		Unlimited range means 'designer solvents'
Polarity			Conventional polarity concepts apply		Polarity concept questionable
Cost			Normally inexpensive		2 to 100 times the cost of organic solvents
Recyclability			Green imperative		Economic imperative
Viscosity/cP			0.2-100		22-40,000
Density/g cm^-3^			0.6-1.7		0.8-3.3
Refractive index			1.3-1.6		1.5-2.2

One of the biggest and challenging industrial concerns is the replacement of volatile organic compounds (VOCs) [50], particularly those that are toxic, such as CH_2_Cl_2_, and those that are highly volatile and flammable, such as ethers (Table 1). The use of ILs [51] to replace or reduce VOCs is a move that could have significant positive environmental impact. There would also be safety benefits resulting from the preferential use of ILs over VOCs, largely due to their low or non-flammability with normal use [52,53,54]. To date, the needed information on the environmental outcome, any potential toxicity, stability issues and others for most of the ionic liquids are not yet fully made available and thus they should be treated with caution during their applications.

The uniqueness of structural tunability of both the cations and anions suggests great flexibility in the potential applications of ionic liquids. For example, the melting points of 1-alkyl-3-methylimidazolium tetrafluoroborates [55] and hexafluorophosphates [40] are a function of the length of the 1-alkyl group, which forms a liquid crystalline phase for alkyl chain length over 12 carbon atoms. Another important property that changes with structure is the miscibility of water in these ionic liquids. For example, 1-alkyl-3-methylimidazolium tetrafluoroborate salts are miscible with water at 25 °C where the alkyl chain length is less than six, but at or above six carbon atoms, they form a separate phase when mixed with water. This behavior can be of substantial benefit when carrying out solvent extractions or product separations, as the relative solubilities of the ionic and extraction phase can be adjusted to make the separation as easy as possible. Hence, a tremendous amount of work has been carried out on applications of ionic liquids and has stimulated interest in both academia and industry [1,2,3,4,5,6,56].

**Scheme 1:**
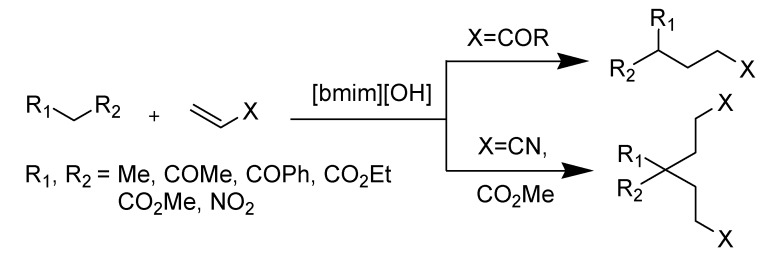
IL-mediated Michael addition reaction in ionic liquid.

Recent development of ILs turned on designing suitable ionic liquids with specific application repetition of ILs many times that can be used both as catalysts/promoters and solvents. Several innovative synthetic procedures on this task lead to successful results and emerge as a new field in ionic liquid chemistry. For example, Ranu *et al*. explored the influence of a new tailor-made, task-specific ionic liquid like [bmim][OH] on Michael addition reactions [57]. This IL, that acts as both catalyst and solvent, plays a dramatic role in the chemical transformation (Scheme 1). Several reviews related to this type ILs are available and, therefore, this subject is not included as a part of our review work.

**Scheme 2:**
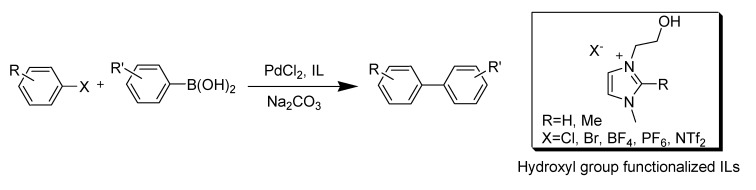
Functionalized ILs used efficiently for the Suzuki coupling reaction.

Dyson *et al*. studied the multi-role of the hydroxyl group in hydroxyl-functionalized ILs (Scheme 2). This IL not only acts as a solvent but also can be viewed as a promoter and demonstrates that careful choice/design of the solvent can be used to tune catalytic reactions [58]. This type of designed functionalized ILs is beyond the scope of this review and will not be discussed here.

## 3. Chemical Stability of Ionic Liquids

### 3.1. Imidazolium ionic liquids

**Scheme 3:**
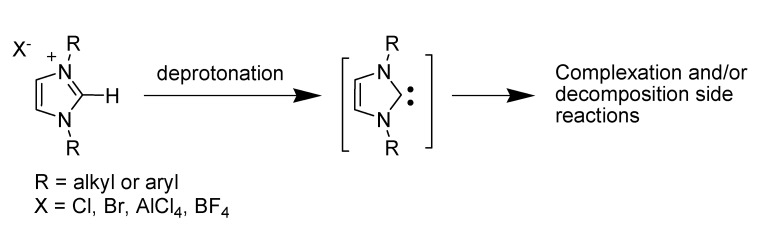
The non-innocent nature of imidazolium-based ILs.

Among RTILs, those derived from imidazole continue to attract increasing interest in many different areas of chemistry [7,8,9,10,11,56]. The imidazolium cation has earned a great attention among scientists due to its low melting points and viscosity, ease of synthesis, and good stability to oxidative and reductive conditions [59]. RTILs are, in general, viewed solely as solvents and, in recent days, its non-innocent nature however began to emerge (Scheme 3). Deprotonation affording *N*-heterocyclic carbenes (NHCs), high acidity at C2 position, and easier deuterium exchange have significant implications in the chemistry of this type of ILs [60].

Noting these properties, it becomes immediately obvious that imidazolium-based ILs are likely to be chemically unstable under basic conditions and caution must be exercised in interpreting reaction results obtained in such ILs, under basic conditions.

#### 3.1.1. Acidity and deuterium exchange

**Scheme 4:**
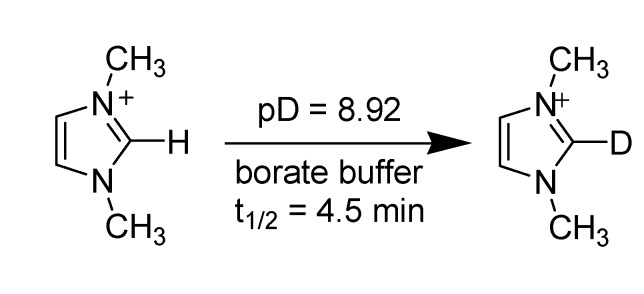
H-D exchange at C2 position of [dmim] cation.

In early 1964, Olofson *et al*. reported the kinetic evidence and study of the rates of deuterium incorporation in heavy water buffers of imidazole related compounds [61]. Their experiments clearly demonstrated the acidic nature of C2 proton of the 1,3-dialkylimidazolium cation, such as 1,3-dimethylimidazolium [dmim], that can be exchanged with deuterium under mild conditions (Scheme 4).

**Scheme 5:**
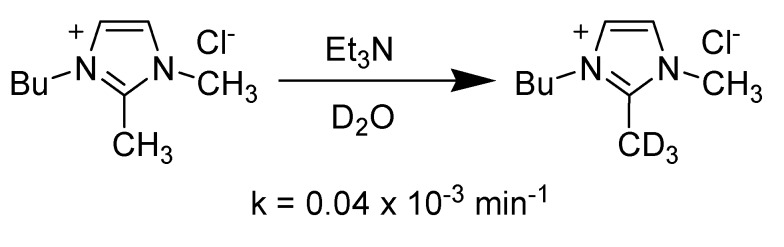
Deuterium isotope exchange at C2 methyl group of [bdmim] cation under basic conditions.

Although substitution at the C2 position of the imidazolium cation prevents the side reaction upon chemical synthesis, Handy and Okello later showed that even the C2 methyl substituted 1-butyl-2,3-dimethylimidazolium [bdmim] cation is not completely inert [62]. They found that the C2 methyl group underwent slow proton exchange even in the presence of a weak base such as triethylamine (Scheme 5).

#### 3.1.2. pKa of C2 hydrogen in imidazolium ions

Since pKa values can reveal the acidic nature of imidazolium cation, several reports determined these values by varying substituents (Table 2). The pKa values of the simple 1,3-dialkylimidazolium cations have been determined in both DMSO [63] and H_2_O [64] and were found to be in the range of 21–24 [63,64,65,66].

**Table 2:** pKa values with different substituents on imidazolium cations.

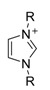			
**Entry**	**R**	**Solvent**	**pKa value**
1	^t^Bu	DMSO	22.7
2	Me	H_2_O	23
3	Me	DMSO	21.1
4	^t^Bu	DMSO	22.6
5	Ph	DMSO	16.1
6	*^i^* Pr(4,5-dimethyl)	DMSO	24

Even though the pKa values of some of these imidazolium cations have been determined, much less attention has been directed towards the impact that the counteranion has on this acidity and exchange at C2 position. Handy and co-workers recently demonstrated that the anion had a strong influence on the conditions required for H-D exchange [62]. Their study revealed that more basic anions such as dicyanimide N(CN)_2_ resulted in RTILs that would undergo deuterium exchange in the absence of any added base (entry 1, Table 3) while weakly coordinating and non-basic anions such as tetrafluoroborate BF_4_ resulted in salts which required an external base for deuterium exchange to occur (entry 2, Table 3).

**Table 3:** Effect of anion on the H-D exchange rate of C2 hydrogen of [bmim] cation.

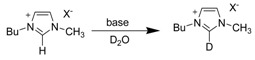			
**Entry**	**Base**	**X**	**Rate (min^-1^)**
1	None	N(CN)_2_	41 x 10^-3^
2	None	BF_4_	0

As aforementioned, the C2 methyl substituted imidazolium ILs also underwent slow proton exchange in the presence of a base. Handy and Okello have shown that even the 2-methyl substituted imidazolium cation is not completely inert (Table 4) [62].

**Table 4:** Effect of anion on the H-D exchange rate of C2 methyl group of [bdmim] cation.inline graphic

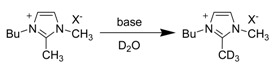			
**Entry**	**Base**	**X**	**Rate (min^-1^)**
1	Et_3_N	Cl	0.04 x 10^-3^
2	Et_3_N	N(CN)_2_	0.04 x 10^-3^
3	KOH	Cl	2.1 x 10^-3^
4	KOH	N(CN)_2_	1.0 x 10^-3^

A distinct influence of the anion on exchange process was reported by Schatz [67]. Very different H-D exchange rates (up to one order of magnitude) could be noted for chloride and bromide salts, depending upon the size of the substituents on the two imidazole nitrogens. The influence of the counterion on the H-D exchange was investigated for compound **1**. In no case, similar rate constants for bromide and chloride were found in methanol-*d*_4_ containing 3% water. The values differ by as much as a factor of 10 (Table 5). For bromides, the exchange rates dropped significantly compared to those of chlorides. However, in the case of bulky substituents, the bromide salts showed higher exchange rates [62].

**Table 5:** Rate constants of the H-D exchange in methanol-*d*_4_ containing 3% water at 300 K. inline graphic

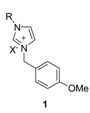			
**Entry**	**R**	**X**	**k (d^-1^)**
1	mesityl	Cl	1.06
2	isopropyl	Cl	0.553
3	tert-butyl	Cl	<< 0.001
4	mesityl	Br	0.176
5	isopropyl	Br	<< 0.001
6	tert-butyl	Br	0.158

Our [b-3C-im][NTf_2_] ionic liquid appears to provide sufficient chemical tolerance to bases so that many potential side reactions that are common in imidazolium-based ionic liquids may therefore be avoided [68]. Under neutral conditions (CD_3_OD/D_2_O = 1:1), ionic liquids such as [bdmim][NTf_2_] and [bdmim][PF_6_] produced less than 10% deuterium exchange at C2 methyl group at room temperature for one week time and only [bmim][PF_6_] was found to be deuterium exchanged rapidly (50% in 1 h) and our [b-3C-im][NTf_2_] gave no detectable exchanges even after one week at ambient temperature [68,69]. Under basic condition (0.1 M KOD in CD_3_OD/D_2_O = 1:1), only [b-3C-im][NTf_2_] ionic liquid is chemically stable to base (t_1/2_ ~ 6 days). Among all tested ionic liquids, [b-3C-im][NTf_2_] ionic liquid was most stable to solvent deuterium isotope exchange while the previously reported [bmim][NTf_2_] and [bdmim][NTf_2_] was deuterium exchangeable instantaneously and in 30 min, respectively, at ambient temperature.

From the results of solvent deuterium exchange experiments, we therefore concluded that the new ionic liquid [b-3C-im][NTf_2_] is far more chemically stable than the previously reported [bmim][PF_6_], [bdmim][NTf_2_], and [bdmim][PF_6_] (i.e., [b-3C-im][NTf_2_] » [bdmim][NTf_2_] ≈ [bdmim][PF_6_] » [bmim][PF_6_]).

#### 3.1.3. Generation and stability of carbenes

**Scheme 6:**
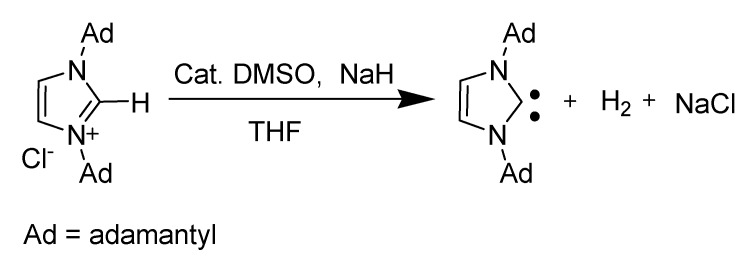
Preparation of a stable NHC.

The characteristic source of reactivity is the high acidity of the C2 hydrogen position in the imidazolium cation [36,37]. Deprotonation of C2 hydrogen results in a highly stabilized *N*-heterocyclic carbene (NHC) (Scheme 6). Initially, NHCs were first proposed by Wanzlick in the 1960s and were characterized by the formation of their adducts with a variety of other compounds [70]. For several years, no other evidence other than these adducts were proposed for NHC formation.

In 1991, Arduengo *et al*. achieved a breakthrough by isolating the stable NHC. By deprotonation of an imidazolium salt, they were able to characterize a stable singlet *N*-heterocyclic carbene, 1,3-di-1-adamantylimidazol-2-ylidene [71,72]. They demonstrated that imidazolium cations with larger groups on the two nitrogens afforded stable isolable NHCs.

**Scheme 7:**
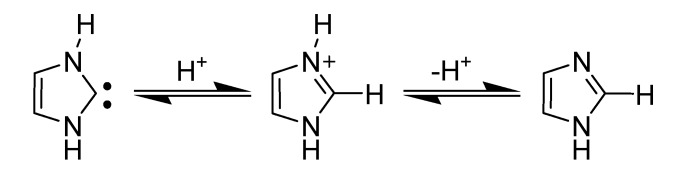
Conversion of NHC to imidazole.

Amyes *et al*. have calculated the equilibrium constant and ∆G^o^ for the conversion of the singlet carbene to neutral imidazole *via* a 1,2-hydrogen shift in water to be 5×10^16^ and -22.7 kcal/mol, respectively [73]. The concerted 1,2-shift is however symmetry forbidden and hence the reaction to give imidazole occurs in two steps as shown in Scheme 7.

The first experimental measurements of gas phase proton affinity (251.3 ± 4 kcal/mol) of the *N*-heterocyclic carbene, 1-ethyl-3-methyl-imidazol-2-ylidene **2**, lead to greater insight into their reactivity [74].


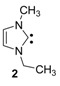


**Scheme 8:**
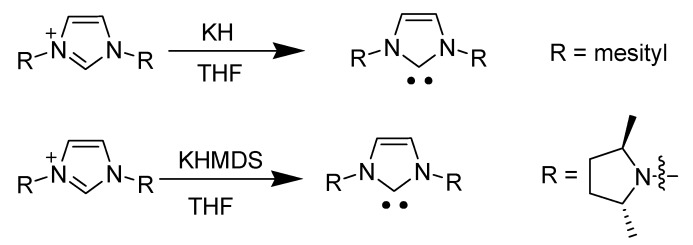
Common preparation of NHCs.

Preparations of NHCs from imidazolium salts usually involve the use of strong bases such as potassium or sodium hydride, LDA or KHMDS (Scheme 8) [75,76].

#### 3.1.4. Reactions involving C-C bond formation

##### 3.1.4.1. Baylis-Hillman reaction

**Scheme 9:**
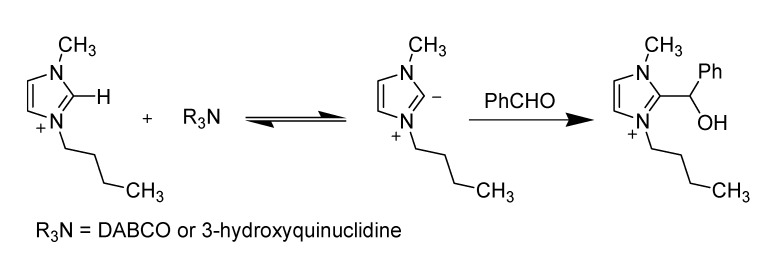
Nucleophilic addition of [bmim] cation onto benzaldehyde.

In 2001, Afonso *et al*. anticipated that imidazolium-based ionic liquids would be a good choice of solvent for the Baylis–Hillman reaction [77]. As they assumed, the reaction was “found” to be 33 times faster in [bmim][PF_6_] than in acetonitrile. The reactions were, however, low yielding. Soon after Afonso’s paper was published, Aggarwal *et al*. quickly pointed out that the ionic liquids used were all [bmim]-based and, in the presence of bases such as DABCO or 3-hydroxyquinuclidine, the imidazolium moiety in ionic liquids could be deprotonated at its C2 position and the resulting nucleophile directly reacted with and thereby consumed the aldehyde (Scheme 9), leading to the misinterpretation of both the apparent reaction rates and the lower yields [78,79].

**Scheme 10:**
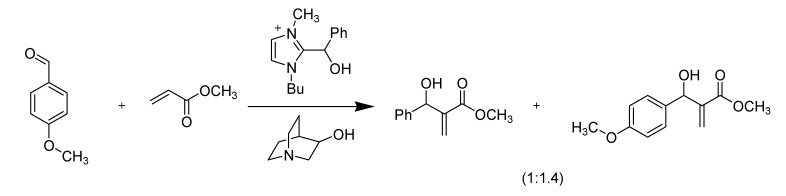
Mixed Baylis-Hillman adducts result if [bmim]-based ionic liquid was recycled for new reaction with different starting aldehyde.

Further, they also showed that caution must be exercised when using ionic liquids recycled from one reaction to another. If the adduct formation is reversible as in Scheme 9, the ionic liquid recycled from one run to another of a Baylis-Hillman reaction provided a mixture of products (Scheme 10).

Our group made a comparative study on the use of [bmim][PF_6_] and [bdmim][PF_6_] in the Baylis-Hillman reaction and demonstrated that the product was contaminated with aldehyde-conjugated [bmim][PF_6_] when this IL was used [80].

Itoh *et al*. also reported that no reaction took place when PhMgBr was reacted with benzaldehyde in conventional [bmim][NTf_2_] ionic liquid and the desired product was obtained for the [bdmim][NTf_2_] solvent system [81]. These results clearly demonstrated once again that the strong acidity of the C2 proton of the imidazolium salt caused decomposition of the Grignard reagents [82,83].

Handy *et al*. carried out alkylation reactions of [bmim][Br] with various alkyl halides using the base NaH *via* generation of carbene intermediates [84]. Their results revealed that primary alkyl halides (butyl, hexyl, heptyl, decyl, and hexadecyl), except allyl bromide, provided 2-substituted imidazolium RTILs, while secondary alkyl halides failed to do so. They also showed that 2-substituted imidazolium could be obtained as a sole product by using excess NaH [62].

**Scheme 11:**
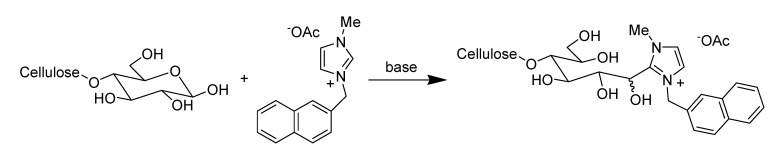
Reaction of 1-methyl-3-(2-naphthylmethyl)imidazolium acetate ionic liquid with cellulose at its reducing end.

Most recently, Rosenau *et al*. demonstrated unambiguously that 1-alkyl-3-methylimidazolium ILs react at C2 with the reducing ends of cellulose and aldopyranose models [85]. They found the reaction was strongly catalyzed by bases such as imidazole and 1-methylimidazole, commonly present impurities in ILs. In solutions of cellulose in 1-alkyl-3-methylimidazolium ILs, both the ionic liquid and the cellulose are evidently not inert (Scheme 11). This again confirmed that C2 position of imidazolium was chemically unstable under basic conditions.

This might induce adverse effects in medical and biological applications if even minor impurities present in cellulosics and cellulose derivatives. The application of alkylmethylimidazolium ILs in the processing of oxidized cellulosics, such as TEMPO-oxidized or periodate-oxidized cellulose, seems to be rather problematic, for medical and physiological scenarios.

##### 3.1.4.2. Palladium catalysed reactions

**Scheme 12:**
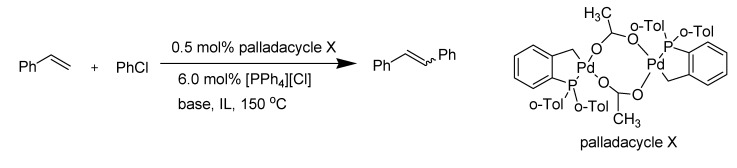
Heck reaction forming palladacycle.

*Heck Reaction:* Carmichael *et al*. reported that ionic liquids provide a convenient medium for the Heck reaction, while allowing recycling of the catalyst [86]. Herrmann and Böhm found that the imidazolium-based ionic liquids gave less satisfactory results compared to quaternary ammonium salts (Scheme 12, Table 6) [87,88].

**Table 6:** Heck reaction of chlorobenzene and styrene in presence of palladacycle X^a^.

IL	Base	Time (h)	Yield (%)^b^
[N_4,4,4,4_][Br]	NaOAc	18	51
[pmim][Br]	NaOAc	19	22
[pbim][Br]	NaOAc	16	11
[bbim][PF_6_]	NaOAc	15	5

^a^ Conditions: 1.0 equiv of chlorobenzene, 1.5 equiv styrene, 1.2 equiv base; ^b^ GC yields.

**Scheme 13:**
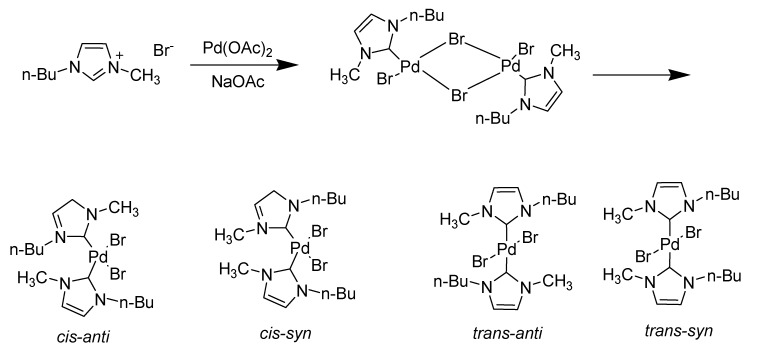
Catalytic active [bmim][Br] for olefination of aryl halides.

This observation suggested that the Heck reaction when carried out in imidazolium ionic liquids could proceed through a different mechanism *via* formation of carbene species. Xu *et al*. obtained the first convincing evidence that the imidazolium ion can react with a catalyst precursor to form NHCs [89]. They also clearly showed that the carbene complex of palladium formed between Pd(OAc)_2_ and the solvent [bmim][Br] is catalytically active for olefination reactions of aryl halides (C-C bond formation reaction) (Scheme 13) [90].

In order to determine the effect of the ionic liquid, a control study on these reactions was performed. Like in the Heck reaction, Pd(OAc)_2_ was heated in [bmim][Br] in the absence of the alkene and aryl halide, under reaction conditions. They observed rapid color changes from dark brown to red and then to yellow and the products formed were analyzed by NMR. The characteristic chemical shifts of the olefin protons (δ 7 in the ^1^H-NMR spectrum) and the carbene carbons (δ 160–170 in the ^13^C- NMR spectrum) suggested the presence of a mixture of palladium carbene complexes. Supporting this formation of Pd carbene complexes, other species such as Ir carbene complexes (as well as hydrogenated species) are also obtained in reactions with imidazolium salts [91].

**Scheme 14:**
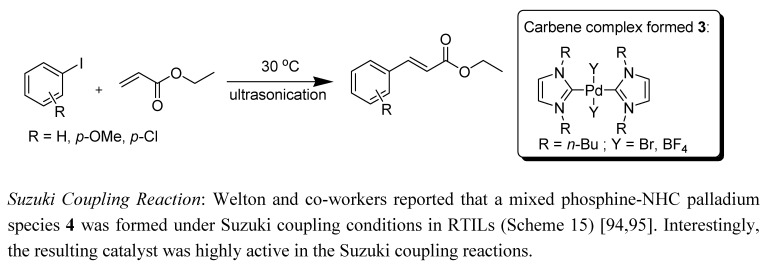
Heck reaction *via* ultrasonic irradiation with IL.

McGuiness *et al*. proposed a mechanism for the Heck reaction catalysed by Pd-carbene complexes [92]. The active species is the 14-electron Pd(0) complex Pd(tmiy)_2_ to which the oxidative addition of an aryl halide occurs, giving the Pd(II) aryl complex. Dissociation of the halide ligand is accompanied by olefin coordination, insertion, and *β*-hydride elimination to release the product and give (after recoordination of the halide) Pd(tmiy)_2_HX. Reductive elimination of HX in the presence of base then regenerates the active Pd(0) species. Srinivasan *et al*. have performed the Heck reaction at ambient temperature with considerably enhanced reaction rates by the combined use of ultrasonic irradiation and ionic liquids as solvents (Scheme 14) [93]. They confirmed the formation of the carbene complex **3** by ^1^H-NMR analysis.

**Scheme 15:**
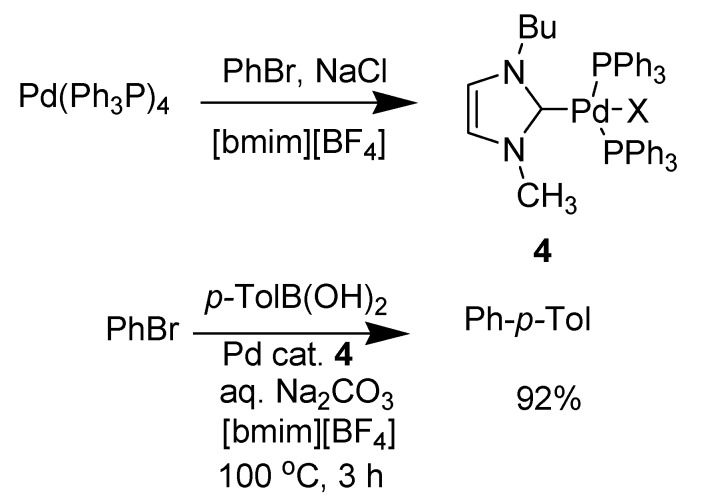
Suzuki coupling reaction with RTILs.

Mathews *et al*. had previously demonstrated the *in situ* formation of a mixed phosphine-imidazolylidene palladium complex in the ionic liquid [bmim][BF_4_] [94]. Wong *et al.* [96] carried out the Suzuki cross-coupling of 4-bromoacetophenone (BrAP) and phenylboronic acid (PBA) catalysed by tris(dibenzylideneacetone) dipalladium chloroform complex (Pd_2_*(*dba*)*_3_–CHCl_3_) which showed the dramatic difference in yields in different solvents.

**Scheme 16:**
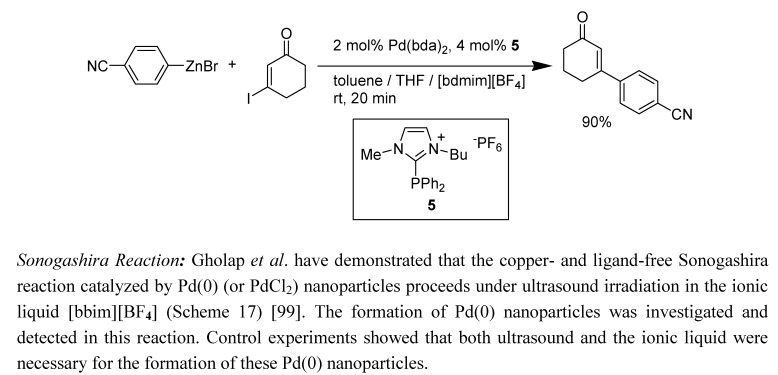
Negishi cross-coupling of an organozinc in ionic liquid.

*Negishi Cross-Coupling Reaction*: Obviously, if deprotonation of the imidazolium cation with an amine base can be a problem for organic chemists, use of nucleophilic organometallic reagents such as Grignard, organolithium, and even organozincs should certainly be more problematic. Indeed, the use of [bmim]-based RTILs in organozinc catalysed Negishi couplings met with failure; on the other hand, when a new ionic phosphine ligand **5** was incorporated for the reaction performed in a biphasic solvent system of toluene and [bdmim][BF_4_], this palladium catalyzed cross-coupling reaction proceeded smoothly giving high isolated yields (Scheme 16) [97]. Chan and co-workers attempted a reaction of diethyl zinc with carbonyl compounds in the ionic liquid [bmim][Br], in which they observed gas evolution and formation of NHC-zinc complexes [98].

**Scheme 17:**
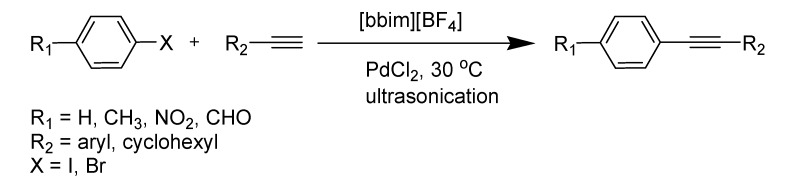
Sonogashira reaction by Pd nanoparticles in IL.

*Sonogashira Reaction:* Gholap *et al*. have demonstrated that the copper- and ligand-free Sonogashira reaction catalyzed by Pd(0) (or PdCl_2_) nanoparticles proceeds under ultrasound irradiation in the ionic liquid [bbim][BF_4_] (Scheme 17) [99]. The formation of Pd(0) nanoparticles was investigated and detected in this reaction. Control experiments showed that both ultrasound and the ionic liquid were necessary for the formation of these Pd(0) nanoparticles.

Magna *et al*. investigated the telomerization of butadiene with methanol and clearly demonstrated the existence of reactivity between the Pd catalyst and 1,3-dialkylimidazolium ionic liquids [100]. They believed that this indicates a stoichiometric reactivity between 1,3-dialkylimidazolium salt and palladium, leading eventually to an inactive form. The putative formation of a “carbene-Pd” complex was evoked to explain the inactivity of systems using this kind of ionic liquids.

*Aromatic Substitution Reactions:* Earle *et al.* have recently reported that certain ionic liquids are chemically reactive and can greatly influence the outcome of the electrophilic aromatic substitution reactions [101]. Lancaster *et al.* investigated the aromatic nitration in various ionic liquids with a range of nitrate salts and explained the importance of cation choice among ILs that can influence the selectivity, yield, and reaction time (Table 7) [102]. They observed low yields when using [bmim] and [bdmim] compared to [bmpy] ILs. This might be due to the stability of the ionic liquids towards the nitrating agent used. Their MS analysis revealed that one or two nitro group substitutions were observed in [bmim] and [bdmim].

**Table 7:** Electrophilic aromatic nitration of toluene by acyl nitrates in various solvents.

Solvent	System	Time (h)	Yield (%)
CH_2_Cl_2_	HNO_3_ - Ac_2_O	1	35
[bmim][NTf_2_]	HNO_3_ - Ac_2_O	1	42
[bdmim][NTf_2_]	HNO_3_ - Ac_2_O	24	63
[bmpy][NTf2]	HNO_3_ - Ac_2_O	1	93

**Scheme 18:**
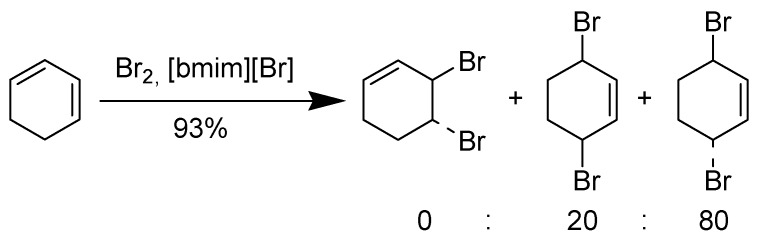
Bromination of 1,3-cyclohexadiene in [bmim][Br] at room temperature.

Chiappe *et al*. have demonstrated the use of ionic liquid solvents for stereoselective halogenations of alkenes and alkynes [103]. Depending on the ionic liquid the addition may be stereospecific, *erythro-* (or *meso-*) bromochlorides or dibromides are obtained from *trans-*olefins while *cis-*olefins give the corresponding *threo-* (or *d,l-*) adducts (Scheme 18).

**Scheme 19:**
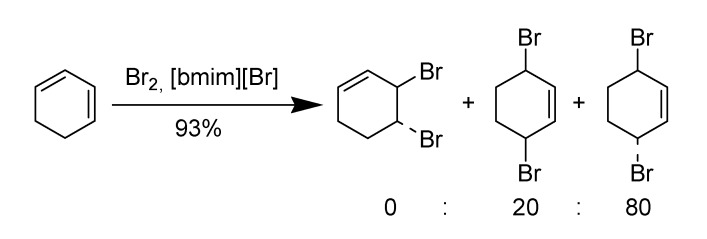
Nucleophilic aromatic substitution in IL.

Our group also found that the nucleophilic aromatic substitution reaction when conducted in the commonly used [bmim][PF_6_] ionic liquid instead of [bdmim][PF_6_], the desired arylamine products 6 were obtained with low yields and found to be contaminated with the 1-fluoro-2-nitrobenzene-conjugated [bmim][PF_6_] adduct 7 (Scheme 19). The chemical nature of adduct could be readily verified and unambiguously confirmed by ^1^H-NMR [104].

Being aware of this acidic incompatibility of imidazolium ionic liquids, our group synthesized and characterized a new class of room temperature ionic liquids [R-3C-im][NTf_2_], which are chemically stable ionic liquids [68,105]. We have also performed high temperature organic reaction such as the Claisen rearrangement in the chemically and thermally stable and non-volatile room temperature ionic liquid [b-3C-im][NTf_2_] under microwave conditions and successfully obtained high isolated yields [106]. Thus, these ionic liquids appear to fulfil practical requirement as an inert solvent for chemical reactions and these ionic liquids may open exciting perspectives of use in various synthetic applications of natural and non-natural products of biological significance. The [R-3C-im][NTf_2_] are the most chemically stable imidazolium-based room temperature ionic liquids available today.

### 3.2. Phosphonium ionic liquids

**Scheme 20:**
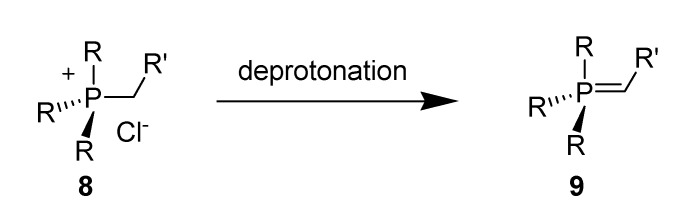
Expected phosphorane product on deprotonation of PIL cation.

Apart from imidazolium ionic liquids, phosphonium ILs have also been investigated with the view of exploring their general scope and their unique capabilities. Phosphonium-based ILs (PILs) are less expensive to manufacture on an industrial scale, and are also an efficient and recyclable media for Pd-mediated cross-coupling reactions [107,108]. PILs are stable under thermal conditions, and strongly basic reagents including Grignard reagents [109,110]. For these reasons they offer greater practicality and scope and deserve far more consideration as unique reaction media in the ILs field than they have received thus far. Clyburne *et al*. [111] found that highly basic NHCs (pKa = 22-24) [63,64] are persistent in PILs [112] such as trihexyl(tetradecyl)phosphonium chloride **8** [107] and were surprised to observe that deprotonation of the IL **8** to produce a phosphorane **9** did not occur (Scheme 20).

Supporting this observation, they also examined whether stronger bases (Grignard reagents) would be persistent and their behaviour in PILs. Interestingly, these solutions again show no sign of degradation after one month, as shown by reactivity studies. The failure of deprotonation of **8** to produce a phosphorane **9** indicates that PIL **8** is capable of supporting reactions involving strong bases such as Grignard reagents without any degradation/reaction between the IL and the strongly basic reagents. They also examined the stability of PIL in the presence of several different nucleophilic reagents. The absence of phosphoranes and phosphoryl in the PILs suggests that they are a suitable choice as solvent for strongly basic, carbon-centered reagents. These observations open up the use of PILs as a reliable reaction media for a wide variety of basic reagents. They also reported that the stability of organometallic species in PILs are anion dependent and evidently shown that trihexyltetradecylphosphoium decanoate would act as solvents for bases such as Grignard reagents, isocyanides and Wittig reagents (phosphoranes) without any decomposition of the solvent [113]. Thus, for phosphonium ions, small bases are susceptible to react with the PIL through deprotonation reactions whereas large bases are resistant to this reaction.

Later, Itoh *et al*. designed new phosphonium salt ionic liquids that are applicable to various types of Grignard reagent mediated reactions [81]. In their study, they demonstrated that the introduction of an alkyl ether moiety on the side arm of phosphonium salt ionic liquid was quite effective in improving the capability of the phosphonium salt ionic liquids as a solvent for ether free Grignard reaction and Grignard reagent mediated reaction. Besides being reported to be sterically hindered at its central core [112,114], lack of acidic ring protons made a belief that commercially available trihexyl-(tetradecyl)phosphonium chloride **8** ionic liquid is chemically stable and remains inert towards Grignard reagents under strongly basic conditions [36,115].

**Scheme 21:**
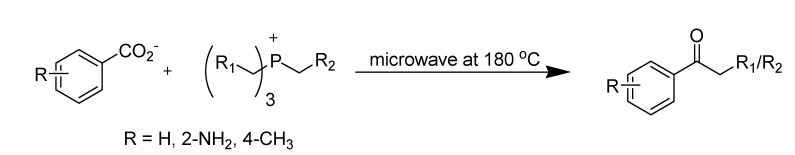
Reaction of benzoate salts with PIL **8**.

Very recently, our group demonstrated the instability of tetraalkylphosphonium ionic liquids [116]. Under basic conditions, **8** was reactive and 50% deuterium exchanged on all four P–CH_2_ methylene groups within 9 h (t_1/2_) at ambient temperature. Further we investigated the direct carboxylate alkylation of organobromides with the presence of Hunig’s base in ionic liquid **8** (Scheme 21).

**Scheme 22:**
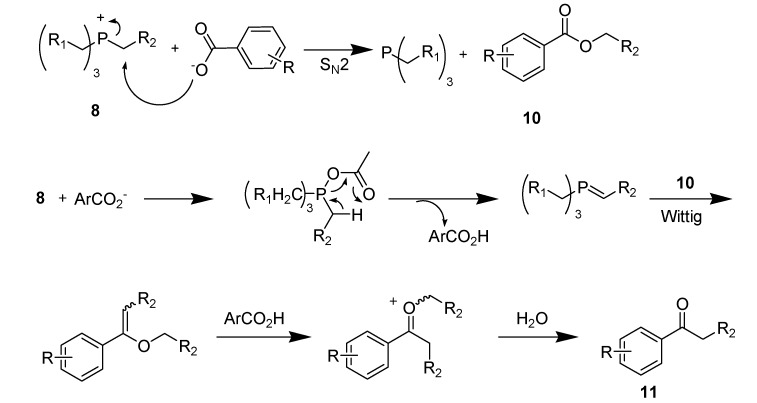
Mechanism proposed for benzoate reaction with PIL **8**.

Our studies quickly revealed that the phosphonium cation reacted with sodium salts of substituted benzoates apparently through direct S_N_2 carboxylate alkylation to form esters **10** and the resulting esters further converted, *via* Wittig reaction, to finally afford aryl ketones **11** by microwave irradiation at elevated temperature. We have also isolated and spectroscopically characterized these aryl ketones. A plausible mechanism was then proposed to account for the fact that aryl ketones **11** are reaction products and benzoate esters **10** are the most probable intermediates (Scheme 22).

Our results revealed for the first time that the commercially available tetraalkylphosphonium cation in ionic liquid **8** is acidic, electrophilic, and can be chemically reactive. Even though phosphonium-based ionic liquids are less prone to thermal decomposition, the presence of O_2_ may lead to the formation of phosphine oxides in many cases [117] and transfer of O from oxygen containing anions, such as HSO_4_^-^, also leads to byproduct formation [118].

### 3.3. Quaternary ammonium ionic liquids

The date of discovery of the "first" ionic liquid starts with the quaternary ammonium ionic liquids. In 1888, ethanolammonium nitrate (mp 52-55 °C) was reported by Gabriel [119]. Later in 1914, one of the earlier known room temperature ionic liquids was [EtNH_3_][NO_3_] (mp 12 °C), synthesized by Walden [120]. These ILs make some unique applications in various fields. Their special properties like low-melting points, low-viscosity, chemical and electrochemical stability facilitate development of ILs as possible safe electrolytes for high-energy density devices [121,122,123,124,125,126].

Calò *et al*. [127] made a comparative study of the effects exerted by different ILs (quaternary ammonium, pyridinium, imidazolium) on catalyst stability, reaction rates and regio- and stereoselectivity in carbon-carbon coupling (Heck, Suzuki, and Stille) reactions. The superiority of quaternary ammonium halides over other ILs might be due to the Coulombic interaction between the cations and anions in ILs as well as the nucleophilicity of the anions. The bulkiness of tetrahedral quaternary ammonium cations, which forces the anions away from the cation, renders these anions available for a good activity and stability of the palladium catalysts. On the contrary, the planar structures of imidazolium and pyridinium cations, due to a strong Coulombic interaction that binds the anions tightly, decrease their availability for stabilisation and activity of the catalysts.

In the Suzuki reaction, quaternary ammonium ionic liquid was less efficient in catalyzing the reaction in presence of inorganic bases. However, the presence of an aqueous base, which is necessary for the Suzuki reaction, accelerated the aggregation of Pd nanoparticles to afford catalytically inactive “Pd black”, which inhibits the catalyst recycling. As previously reported [127,128], whatever the base, these reactions were devoid of stereoselectivity and, yet again, [bmim]-based ionic liquid was less efficient than quaternary ammonium ionic liquid. Similar to earlier reports, the influence of bases on the reaction between 4-bromotoluene and *trans*-ethyl cinnamate catalysed by Pd-benzothiazole carbene complex in quaternary ammonium ionic liquid [129], proved a dramatic role on the regio- and stereoselectivity regardless of the base used.

**Scheme 23:**
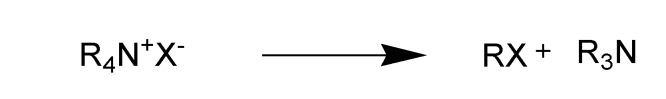
Reverse Menschutkin decomposition in quaternary ammonium ILs.

**Scheme 24:**
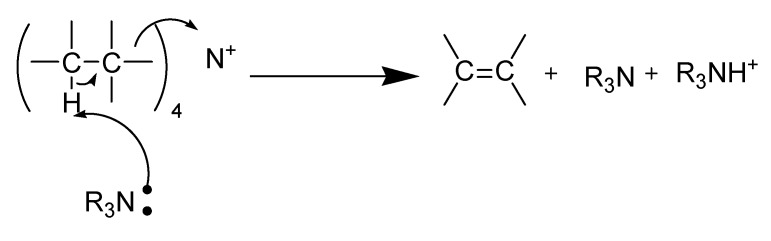
Hofmann elimination reaction in quaternary ammonium ILs.

Two modes of thermal decomposition of quaternary ammonium salt are known [130]: the reverse Menschutkin (Scheme 23) and Hofmann (Scheme 24) types. One would expect reactivity in reverse Menschutkin reaction to follow established orders of X^-^ nucleophilicity toward saturated carbon (Scheme 23).

One might expect reactivity in Hofmann elimination reaction to follow the order of X^-^ basicities (approximately ClO_4_ < I < Br < NO_3_, SCN, picrate) although it is not clear whether bases as weak as the anions can indeed bring about such E2 process [131,132]. However, the equilibrium condition of R_4_N^+^X^-^ with strongly nucleophilic X**^-^** in the fused salt is evidently undergoing complete decomposition to olefin and R_3_NH^+^X^-^ (Scheme 24).

Zhou *et al*. synthesized and characterized cyclic quaternary ammonium salts comprising *N*-alkyl-(alkyl ether)-*N*-methylpyrrolidinium, -oxazolidinium, -piperidinium, and -morpholinium (alkyl = *n*-C_4_H_9_, alkyl ether = CH_3_OCH_2_, CH_3_OCH_2_CH_2_) with a series of weakly coordinating and electrochemically stable fluoro anions, namely perfluoroalkyltrifluoroborate [RFBF_3_]^-^, (RF = CF_3_, C_2_F_5_, *n*-C_3_F_7_, *n-*C_4_F_9_). Some of these salts are potential candidates for use as electrolytes in high-energy storage devices [126].

### 3.4. Pyridinium ionic liquids

The modern era of ionic liquids stems from the work on alkylpyridinium ionic liquids and has various applications. From the literature available for these ILs, it is understood that no systematic and detailed studies on their reactivity and stability have been undertaken.

Magna *et al*. observed a poor regioselectivity when using pyridinium salt in the telomerization of butadiene with methanol, compared to imidazolium salts [133]. This poor regioselectivity was due to the transfer of an alkyl group from pyridinium to triphenylphosphine [134,135], thus producing phosphonium salts, which cannot stabilize Pd(0), thus the appearance of the palladium black.

Strehmel *et al*. revealed that the use of *N*-butyl-4-methylpyridinium salts in free radical polymerization of *n*-butyl methacrylate in ionic liquids tends to result in relatively low degrees of polymerization and very high polydispersities, compared to the analogously substituted 1-alkyl-3-methyl imidazolium tetrafluoroborate and hexafluorophosphate, although the viscosities of 12 was higher [136].


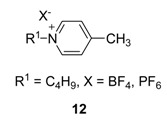


## 4. Instability of Anions in Ionic Liquids

The stability of the anions involved in ionic liquids plays a critical role in certain applications or their toxicity. Typical ionic liquids consist of halogen containing anions such as [AlCl_4_], [PF_6_], [BF_4_], [CF_3_SO_3_] or [(CF_3_SO_2_)_2_N], which in some regard limits their ‘greenness’. The presence of halogen atoms may cause serious concerns if the hydrolytic stability of the anion is poor (e.g., for [AlCl_4_] and [PF_6_]) or if a thermal treatment of spent ionic liquids is required. In both cases, additional effort is needed to avoid the liberation of toxic and corrosive HF or HCl into the environment.

The lower homologues of alkylsulfate anions, namely methanesulfate and ethanesulfate, are known for their sensitivity towards hydrolysis. In the presence of water these anions form the corresponding alcohol and hydrogen sulfate at elevated temperatures. Obviously, this process is undesired for most applications since the ionic liquid system changes dramatically in its properties and an acidic proton is formed that may be a reactive species in many applications. For alkylsulfates with longer alkyl groups the sensitivity towards hydrolysis is much lower [137,138].

Anions present in ILs play a significant role in the property of ionic liquids. The assumption that all ionic liquids are non-coordinating solvents is incorrect. The coordinating property of ILs may also depend on the anion present in that ionic liquid. Ionic liquids containing PF_6_^-^ ions are hydrolytically unstable, have the propensity to decompose and release HPO_2_F_2_, H_2_PO_3_F, H_3_PO_4_, and highly corrosive HF [139]. It is worth mentioning that several ‘uncatalyzed’ reactions reported in [cation][PF_6_] ionic liquids that are, in fact, catalyzed by adventitious HF. Thus, a significant impact of IL anions is the release of HF containing compounds for which considerable care should be taken while using PF_6_ containing ionic liquids. Thus, ILs should be treated with caution due to their unexplored toxicity and/or stability [140]. Rogers *et al*. identified 1-butyl-3-methylimidazolium fluoride hydrate crystallographically as a decomposition product that was obtained from hydrolytic degradation of [bmim][PF_6_] during purification process [141]. They observed the evolution of acidic HF white fumes, which are colorless, highly toxic and corrosive.

Sundermeyer *et al*. designed and synthesised three new fluorinated anions BPFPA, PFTFSI, and PFNFSI related to the most prominent parent BTFSI *via* stepwise substitution of alkylsulfonyl groups by electron withdrawing pentafluorophenyl groups that attracts interests with respect to their use in highly hydrophobic, water immiscible and hydrolytically stable ILs or their use as electrolyte anions such as in lithium salts [142].


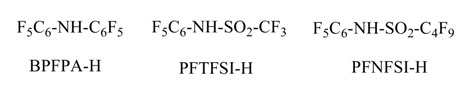

s


**Scheme 25:**
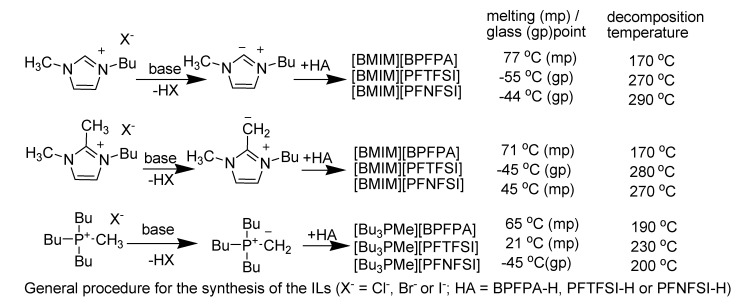
Thermal stability of ILs with fluorinated anions.

The thermal stability of this set of ILs is mainly dependent on the nature of the anion (Scheme 25). Whereas BPFPA derived ILs show a mass loss at temperatures between 170 °C and 190 °C, triflyl and nonaflyl derivatives with PFTFSI and PFNFSI anions exhibit higher thermal stabilities of up to 290 °C . Obviously, this type of ILs is undesired for most applications since the ionic liquid system changes dramatically in its properties and an acidic proton is formed that may be a reactive species in many applications.

## 5. Thermal Stability and Decomposition

**Scheme 26:**
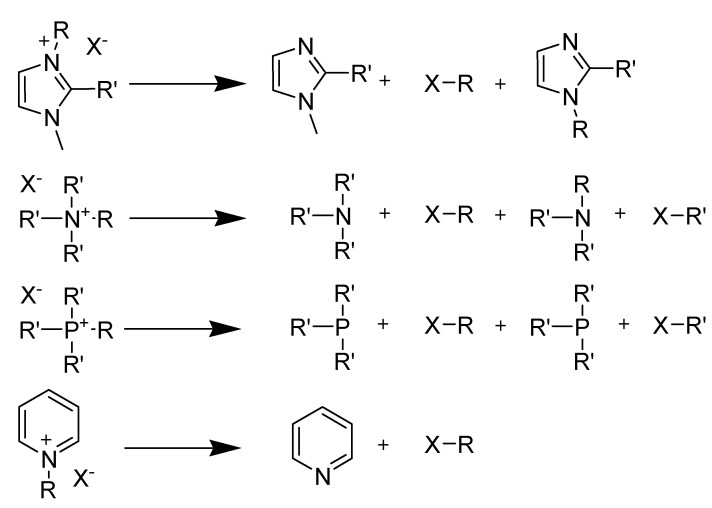
Neutral products formation by nucleophilic attack *via* reverse Menschutkin decomposition.

One of the benefits of ionic liquids, when used as reaction solvents, is their wide liquid range and reportedly high thermal stability [143], which allows application at significantly elevated temperatures in various fields of science. Resistance to degradation (either by intramolecular reaction, reaction between cation and anion or reaction with the atmosphere) is always expected. Most recently, decomposition of some ionic liquids was reported to occur at much lower temperatures than previously reported [144]. A brief summary is the thermal stability of imidazolium and quaternary ammonium ionic liquids is given in this review. Almost in all ionic liquid cases, nucleophilic attack of the ionic liquid anion in a reverse Menschutkin-type reaction to yield neutral products is possible (Scheme 26) [130,145].

It is understood from the literature that phosphonium salts are much more thermally stable than the corresponding ammonium salts and even have an edge on imidazolium salts. This is very important for the processes which operate at temperatures greater than 100 °C. Thermogravimetric analysis suggested that phosphonium ionic liquids were thermally stable up to nearly 400 °C [148]. Moreover, ionic liquids composed of cyano containing anions and cyclic quaternary ammonium cation are prone to polymerisation upon decomposition, while phosphonium-based ionic liquids give only volatile products [149]. Details regarding the thermal stability of phosphoinum and pyridinium ionic liquids are not discussed in this review.

### 5.1. Imidazolium ionic liquids

In general, remarkable differences in *T_d_* are observed by changing the anions, while a simple extension of alkyl chain hardly affects the *T_d_* in imidazolium cation. ILs composed of BF_4_^-^, PF_6_^-^, NTf_2_^-^, are thermally more stable than corresponding halides. The relative anion stability follows the order: PF_6_ > BF_4_ > AsF_6_ >> I, Br, Cl (Table 8). Among anions, inorganic anions undergo endothermic thermal decomposition whereas organic anions like (C_2_F_5_SO_2_)_2_N^-^ and (CF_3_SO_2_)_2_N^-^ generally restricts its exothermic decomposition up to 350 °C. This exothermicity of organic anions is likely to be a consequence of the sulfonyl groups.

**Table 8:** Range of *T_d_* for some imidazolium ILs [146,147,148,149,150].

Ionic liquids	T_d_ range (°C)
[bmim][dca], [bmim][Cl], [bmim][Br], [bmim][I], [Bnmim][Cl], [C_3_mim][Cl], [bmim][Cl],	240-280
[C_5_mim][Cl], [eC_3_mim][I], [mC_2_mim][Cl], [mC_3_mim][Cl], [mC_4_mim][Cl], [mBnmim][Cl]	
[bmim][PF_6_], [bmim][dca], [eC_3_mim][I], [mBnmim][BF_4_]	281-320
[bmim] [BF_4_], [bmim] [OTf], [bdmim] [PF_6_], [bdmim][BF_4_], [C_3_mim] [BF_4_], [bmim][BF_4_],	
[Bnmim][BF_4_], [decmim][BPh_4_], [eC_3_mim][BF_4_], [emim][PF_6_], [mC_2_mim][BF_4_],	360-400
[mC_3_mim][BF_4_], [mC_4_mim][BF_4_]	
[C_5_mim][BF_4_], [bmim][methide], [bmim][NTf_2_], [emim][BF_4_], [pmmim][NTf_2_], [emim][NTf_2_]	401-450
[bdmim][N_3_], [C4mim]_2_ [ZnBr_2_Cl_2_], [pmim][NTf_2_]	451-520

A thermolysis study of dialkylimidazolium salts reports that the decomposition is a S_N_2 process that results in a mixture of *N*-alkylimidazoles and 1-alkylhalides [151]. Ohtani *et al*. studied the thermal decomposition behaviours of several imidazolium based ionic liquids by pyrolysis-gas chromatography [152]. They observed that the thermal decomposition proceeds through C-N bond cleavage. As for the imidazolium halides, corresponding haloalkanes and 1-alkylimidazoles were predominantly formed through nucleophilic attack of halide anions to the alkyl groups, which could preferably occur at methyl groups probably due to a steric effect. Meanwhile, imidazole rings did not decompose under the experimental conditions at around 550 °C.

Suslick *et al*. have investigated the stability of a variety of ionic liquids such as [bmim][Cl], [bmim][BF_4_], [bmim][PF_6_], urea ammonium nitrate (UAN), and decylmethylimidazolium tetraphenyl-borate under ultrasound conditions and reported that, upon sonication, all of the imidazolium ionic liquids darkened from colorless to amber while UAN did not undergo a color change [153]. ^1^H-NMR analysis of [bmim][Cl] and [bmim][BF_4_] also indicated the appearance of new peaks in the imidazole region. Imidazolium ionic liquids produced gases/vapors that contained trace amounts of light hydrocarbons and nitriles consistent with the decomposition of imidazoles. Thus, the ionic liquids do decompose under ultrasonic conditions.

### 5.2. Quaternary ammonium ionic liquids

*T_d_* of quaternary ammonium ILs give a wide range for a particular pair of cation and anion (Table 9).

**Table 9:** Range of *T_d_* for some quaternary ammonium ILs [150].

Entry	Ionic liquid	T_d_ range (^o^ C)
1	[NH_4_][NO_3_]	160
2	[TMA][BF_4_]	688-808
3	[TEA][BF_4_]	663-745
4	[TPA][BF_4_]	605-710
5	[TBA][BF_4_]	598-705

Previously, Sheikh studied the thermal decomposition of R_4_NBX_4_, where R is ethyl and X are C1, Br, and phenyl dichloride [154]. Prasad *et al*. studied the onset temperatures of thermal decomposition and showed that, as the size of the substituted alkyl group increases, the thermal stability of these compounds decreases [150]. It is well accepted that quaternary ammonium compounds of the type R_4_N^+^X^-^ decompose thermally to yield an amine (R_3_N) and the corresponding alkyl compound. In a prelude to the study of the effects of R_4_N^+^X^-^ on ammonium perchlorate (AP, an oxidant) decomposition, they interpreted that the amine (R_3_N) formed by decomposition of IL can interact with the HCIO_4_ produced during the thermal decomposition of AP, thus forming an amine perchlorate [155,156].

### 5.3. Decomposition

As organic salts, ionic liquids are prone to oxidative degradation, particularly with vigorous oxidizing agents has been demonstrated by Pernak *et al*. with KMnO_4_ [157] and O_3_ [158]. They observed that the cations were ultimately oxidized to CO_2_ and H_2_O, while the anions were not oxidized. They observed that ILs undergo decomposition under the effect of ozone in aqueous solution more efficiently. Dynamically growing application of ILs induces misgivings as to their utilization when the real effects of cations and anions are unrevealed.

Shkrob *et al*. reported that the radiation stability from pulse radiolysis studies indicate that in ILs composed of aromatic cations (such as imidazolium and pyridinium), the electrons rapidly attach to the ring to form the corresponding neutral radicals [159]. In another intentional degradation study, Stepnowski and Zaleska demonstrated that imidazolium and pyridinium ionic liquids are degraded by a combination of UV radiation and photocatalysis [160].

## 6. Conclusions

Our review emphasizes the urgent need for understanding the fundamental chemistry of ionic liquids as their incompatibility is now being revealed gradually. Most repeated justifications that ILs are inert and do not interact with the reagents is only an assumption until its properties are clearly studied. Unique and hidden chemistry of ILs limits their application in certan synthetic reactions. ILs, like all other solvents are chemicals which have intrinsic reactivity and thus the acidic nature, instability of anions, thermal stability, and degradation with oxidising agents has always to be kept in mind while choosing ionic liquids as reaction media.
